# Strong population genetic structure of an invasive species, *Rhynchophorus ferrugineus* (Olivier), in southern China

**DOI:** 10.1002/ece3.3599

**Published:** 2017-11-07

**Authors:** Guihua Wang, Youming Hou, Xiang Zhang, Jie Zhang, Jinlei Li, Zhiming Chen

**Affiliations:** ^1^ State Key Laboratory of Ecological Pest Control for Fujian and Taiwan Crops Fujian Agriculture and Forestry University Fuzhou China; ^2^ Fujian Province Key Laboratory of Insect Ecology College of Plant Protection Fujian Agriculture and Forestry University Fuzhou China; ^3^ Fuzhou Entry‐Exit Inspection & Quarantine Bureau of P.R.C. Fuzhou China

**Keywords:** anthropogenic activities, gene flow, genetic structure, geographical distance, invasive pest, *Rhynchophorus ferrugineus*

## Abstract

The red palm weevil (RPW), *Rhynchophorus ferrugineus* (Olivier), was initially reported in China in the 1990s and is now considered one of the most successful invasive pests of palm plants in the country. A total of 14 microsatellite loci and one mitochondrial cytochrome oxidase subunit Ι (*cox I*) gene fragment were used to investigate the genetic characteristics and structure of *R. ferrugineus* in southern China. High levels of genetic differentiation among populations and significant correlations between genetic and geographical distances indicated an important role of geographical distance in the distribution of the RPW in southern China. High gene flow between Fujian and Taiwan province populations illustrated the increased effects of frequent anthropogenic activities on gene flow between them. Genetic similarity (i.e., haplotype similarity) indicated that RPW individuals from Taiwan and Fujian invaded from a different source than those from Hainan. To some extent, the genetic structure of the RPW in southern China correlated well with the geographic origins of this pest. We propose that geographical distance, anthropogenic activities, and the biological attributes of this pest are responsible for the distribution pattern of the RPW in southern China. The phylogenetic analysis suggests that the most likely native sources of the RPW in southern China are India, the Philippines, and Vietnam.

## 1 INTRODUCTION

Invasive species colonizing new environments not only have an economic impact but also often have a negative effect on biodiversity (Sakai et al., 2001). Genetic variation and population genetic structure are closely related to the evolutionary potential of a species and its resistance to a hostile environment (Grant, 1991; Hughes & Dorn, 2002; Nei, 1978). Characterizing the current population genetic structure of an invasive species can facilitate the identification of the source of the infestation, possible invasion paths, and genetic consequences of invasion (Bock et al., 2015). Genetic studies of invading pest species are crucial to understanding how organisms are able to successfully adapt to and colonize new environments.

The red palm weevil (RPW), *Rhynchophorus ferrugineus* (Olivier) (Coleoptera: Curculionidae), a serious invasive palm pest with origins in south‐eastern Asia and Melanesia (Abraham, Koya, & Kurian, 1975; Murphy & Briscoe, 1999), was initially described in India in 1891 (Lefroy, 1906; Vidyasagar, 1998). A wide range of climates, coupled with intensive modern‐date palm farming, has provided the pest an ideal ecological habitat (Abraham, Al‐Shuaibi, Faleiro, Abozuhairah, & Vidyasagar, 1998). This pest has recently been widely introduced to several other areas, including the Middle East, Mediterranean countries (Bozbuga & Hazir, 2008; Faghih, 1996; Gomez & Ferry, 1999), North Africa, Europe, the Caribbean, Oceania, North America (Borchert, 2009; EPPO, 2008; Murphy & Briscoe, 1999), and southern China (Hou, Wu, & Wang, 2011; Ju et al., 2006; Qin, Li, & Huang, 2009; Wan, Hou, & Jiang, 2015). The RPW has a broad host range, from four species reported in 1956 to 40 palm species as well as two nonpalm hosts (*Agave americana* and *Saccharum officinarum*) reported in 2013 (Anonymous, 2013; Esteban‐Duran, Yela, Crespo, & Alvarez, 1998; Lever, 1969; Nirula, 1956; OJEU, 2008). Plants that are less than 20 years old appear to be especially susceptible (Abraham et al., 1998; Nirula, 1956) because females prefer to lay their eggs on younger plants and because these plants provide enough food for larvae. The high reproductive capabilities of the adults and the serious damage caused by the larvae within the trunks make management of this pest particularly difficult.

The exact time that the RPW invaded China is unknown. It was not until the 1990s that scientists began to pay attention to this pest. In southern China, ornamental palm plants are widely used as roadside shade trees, especially in some tourist cities. The first detection of the RPW in Guangdong Province (Zhongshan City) was reported in 1997 (Liu, Zhao, Xu, Chen, & Huang, 2009). This pest was later detected in Hainan (first detected in Wenchang City) in 1998 and rapidly spread across the whole island (Liu, Peng, & Fu, 2002; Wu & Yu, 2001). Due to palm plant transportation, the RPW has been widely dispersed throughout southern China over the last thirty years (He, Yu, Chen, Zhang, & Chen, 2013; Hu, 2006; Li & Yao, 2006; Liu, 2007; Xu, 2005; Zhang, 2007). Considering the serious economic impact and the ecological destruction caused by this weevil, a better understanding of its genetic diversity and of the relationships among different geographical populations is necessary for the development of effective pest management strategies. Various factors, such as geographical barriers, climate conditions, and historical processes, as well as the dispersal abilities of the species, would influence the population genetic structure of an organism (Sun, Lian, Navajas, & Hong, 2012). RPW adults rely on flight for their short‐distance dispersal but can also be carried for long distances by human activities (Qin et al., 2009), which may result in a complex population structure. Furthermore, great distances or geographical barriers are major factors that can prevent gene flow between populations, leading to genetic divergence (Campbell, Mrazek, & Karlin, 1999). There are two straits located in southern China that are likely to influence the distribution of this pest: Taiwan Strait and Qiongzhou Strait. Therefore, special geographical locations and frequent urban landscape construction increase the possibility of RPW dispersal over a wider range in southern China. We hypothesize that large geographical distances combined with frequent human activities may interact to affect the structure of RPW population genetics. Examining how dispersal or movement patterns govern the genetic structure of this pest can help to better understand its ability to colonize rapidly.

The genetic variation of the RPW has been studied in most infested areas, including the Middle East, the Mediterranean Basin, and South Asia (Abulyazid, Kamel, Sharawi, & El‐Bermawi, 2002; El‐Mergawy, Nasr, Abdallah, & Silvain, 2011; Rugman‐Jones, Hoddle, Hoddle, & Stouthamer, 2013). High genetic similarity was found between the RPW populations from the Kingdom of Saudi Arabia (KSA) and those from Indonesia, while no similarity was detected between the Egyptian and KSA or Indonesian populations (Abulyazid et al., 2002). Genetic variation studies of the RPW in the Middle East and the Mediterranean Basin using the *cox I* gene revealed that RPW populations can be subdivided into different subpopulations under the influence of genetic drift favored by founder events following three different invasive routes over 30 years (El‐Mergawy et al., 2011). Anthropogenic activities were considered one of the important factors affecting the distribution of the RPW (Qin et al., 2009). Rugman‐Jones et al. (2013) investigated the large‐scale genetic structure of the RPW over the distribution areas. Although there have been many studies about the RPW conducted around the world, fewer studies have focused on the genetics of the RPW in China, which is adjacent to the native range of this pest. In this study, samples were obtained from a total of eight infested locations in southern China, and the samples were analyzed to describe the genetic structure of the RPW and to determine genetic differentiation among populations using microsatellite loci and *cox I* sequences. Elucidating the population genetic structures of this pest will enhance our understanding of its invasion pathways and subsequently aid in developing an effective control strategy for the RPW in southern China.

## 2 MATERIALS AND METHODS

### 2.1 Samples and DNA extraction

A total of 112 RPW adults were collected from palms in different areas of southern China during 2013 and 2014 (Table S1). Specimens were preserved in absolute ethanol and stored at −20°C. Total genomic DNA extraction and DNA quality detection were performed according to Wang, Zhang, Hou, and Tang (2015).

### 2.2 Microsatellite genotyping and *cox I* sequencing

Individuals were genotyped with 14 microsatellite loci (Capdevielle‐Dulac, 2011, personal communication, Table S2). Forward primers were labeled at the 5′ end with fluorescent dyes (FAM, HEX, or ROX; applied by JieLi Biology, Shanghai, China). The PCR was performed according to Wang et al. (2015). Amplifications were performed with an initial denaturation step of 3 min at 94°C; 40 cycles of 30 s at 94°C, 30 s at the primer‐specific annealing temperature, and 1 min at 72°C; and a final elongation step of 5 min at 72°C.

We amplified and sequenced *cox I* to identify the genetic relationships among the different populations sampled. Forward (5′‐TATAGCATTCCCCGTTTA‐3′) and reverse primers (5′‐TCCTAATAAACCAATTGC‐3′) (modified from Simon et al. (1994)) were used in this study. Cycling conditions were conducted as follows: 94°C for 5 min; 40 cycles at 94°C for 1 min, 48°C for 1 min, and 72°C for 1 min; and a final extension at 72°C for 5 min (El‐Mergawy et al., 2011).

Following PCR product detection by 2% agarose gel electrophoresis and sequencing (Sangon, Shanghai, China), the preliminary sequence analyses were assessed. The allele sizes of microsatellite loci were manually checked and scored based on trace data for further analysis, and mitochondrial PCR products were directly sequenced from both strands with both primers.

### 2.3 Population genetics analysis

#### 2.3.1 Microsatellite data analyses

Because the sample size collected in Sichuan Province was limited and unsuitable for microsatellite analysis, a total of seven populations were genotyped for 14 microsatellite loci. The presence of null alleles was investigated using Micro‐Checker 2.2.3 (van Oosterhout, Hutchinson, Wills, & Shipley, 2004). Genetic diversity statistics, including the mean allele number in each locus (*A*), mean observed heterozygosity (Ho), and expected heterozygosity (He), were estimated using Excel Microsatellite Tools (Park, 2001). Deviations from Hardy‐Weinberg equilibrium (HWE), *F*_IS_ values, and pairwise *F*_ST_ values (Weir & Cockerham, 1984) were calculated using the program GENEPOP (Raymond & Rousset, 1995) (http://genepop.curtin.edu.au/). To minimize the bias of genetic diversity statistics affected by null alleles, genotypes were corrected based on the allele frequencies by the EM algorithm using FreeNA (Dempster, Laird, & Rubin, 1977). *F*_ST_ values were corrected for null alleles present at microsatellite loci by implementing the ENA algorithm within the program FreeNA (Chapuis & Estoup, 2007). Isolation by distance (IBD) using *F*_ST_/(1−*F*_ST_) and the semi‐logarithmic geographical distance matrix (km) among all populations were determined by GENEPOP. The mean allelic richness (AR) was estimated using the FSTAT program (Goudet, 2001).

BOTTLENECK version 1.2.02 (Cornuet & Luikart, 1996; Piry, Luikart, & Cornuet, 1999) was used to assess deviation from the expected heterozygote excess relative to allelic diversity across 14 loci under different mutation models (two‐phased model of mutation, TPM; stepwise mutation model, SMM; infinite alleles model, IAM). Deviations of observed heterozygosity relative to that expected at drift‐mutation equilibrium were evaluated with the Wilcoxon sign‐rank test with 1,000 iterations (Luikart, Allendorf, Cornuet, & Sherwin, 1998). The population structure of RPW was inferred from microsatellite data using the Bayesian clustering algorithm of STRUCTURE 2.3.3 (Pritchard, Stephens, & Donnelly, 2000), with an initial burn‐in period of 5 × 10^4^ iterations followed by a run of 10^6^ Markov chain Monte Carlo (MCMC) repetitions. We used the admixture ancestry model (Hubisz, Falush, Stephens, & Pritchard, 2009) and the correlated allele frequency model. A series of simulations were run from *K* = 2 to 7 (from *K* = 2 to 12 when microsatellite data of Wang et al., 2015 were included) with 20 replicates for each *K* value. The most likely value of *K* was calculated according to Evanno, Regnaut, and Goudet (2005) in STRUCTURE HARVESTER (Earl & vonHoldt, 2012). DISTRUCT 1.1 (Rosenberg, 2004) was used to graphically display the genetic structure results. The distribution of genetic variance within and among clusters was determined using ARLEQUIN 3.5 (Excoffier & Lischer, 2010) through analysis of molecular variance (AMOVA). Gene flow among populations was estimated using the formula *N*_e_ *m *= (1−*F*_ST_)/4*F*_ST_ (Wright, 1931).

#### 2.3.2 Mitochondrial *cox I* sequence and data analyses

DNA sequence data were derived from 112 samples obtained from eight different locations (see Table S4). All sequences were aligned by CLUSTAL‐X 1.81 (Thompson, Gibson, Plewniak, & Higgins, 1997). The number of haplotypes (Nh), number of private haplotypes (Nph), haplotype diversity (Hd), and nucleotide diversity (π) were calculated using DnaSP 5.10 software (Librado & Rozas, 2009). The pairwise *F*_ST_ values between sample locations were calculated using ARLEQUIN 3.5.

A median joining (MJ) network analysis was performed in NETWORK 4.6.1.1 (Bandelt, Forster, & Röhl, 1999). The maximum‐likelihood method (ML; best model: Kamura 3‐parameter; bootstrap values: 1,000) by MEGA 5.03 (Tamura et al., 2011) was used to construct the phylogenetic tree based on the haplotype data. AMOVA was used to test the genetic differentiation among different clusters by calculating *F*‐statistics from haplotypes with 10,000 permutations using the ARLEQUIN 3.5 software. Mismatch distribution as well as neutrality tests were also calculated using ARLEQUIN 3.5. In addition, a Bayesian skyline plot (BSP) analysis was performed using the BEAST v 2.1.3 program (Bouckaert et al., 2014) to examine the demographic history based on coalescent theory. TRACER v 1.5 (Drummond, Rambaut, Shapiro, & Pybus, 2005) was used to show the results of the BSP analysis.

## 3 RESULTS

### 3.1 Genetic variation

#### 3.1.1 Genetic diversity of microsatellites

The mean numbers of alleles and AR at a microsatellite locus were 3.74 and 3.32, respectively. The results showed that He and Ho ranged from 0.273 to 0.650 and from 0.250 to 0.621, respectively. Null alleles were observed at five microsatellite loci with a frequency ranging from 0.022 to 0.283. Null alleles existed in the P1F8 locus in Fujian and Hainan populations; both P1E2 and P2F11 showed null alleles in the GXNN, GDSZ, HNWC1, and HNWC2 populations, and the markers P1C8 and P3A8 showed null alleles in the GXNN and GDSZ populations (data not shown). After correcting the data set for null alleles using the EM algorithm, most of the expected and observed heterozygosity values were slightly higher than the raw data, ranging from 0.273 to 0.660 and 0.250 to 0.677, respectively, with the lowest expected and observed heterozygosity values in the TWTZ population and the highest in the GXNN population (for genetic variability details, see Table 1). Deviations from HWE were observed in four populations (the GXNN, HNWC1, HNWC2, and GDSZ populations) in both raw data and corrected data. The average fixation index (*F*_IS_), which relates to the inbreeding level in each population, produced positive values in all of the populations except the GXNN population under the corrected data. Nonsignificant heterozygote deficiency was observed in the GXNN population (*p *>* *.05) (data not shown).

**Table 1:** Genetic diversity measures estimated using microsatellites and the *cox I* gene of *Rhynchophorus ferrugineus* populations

Pop	*A*	AR	^R^Ho/^C^Ho	^R^He/^C^He	^R^F_IS_/^C^F_IS_	P (HEW)	Nh	Nph	Hd	π (%)	*k*
FJTA	2.20	2.18	0.264/0.280	0.320/0.330	0.140/0.172	ns	3	1	0.417	0.858	4.667
FJSM	2.86	2.77	0.287/0.302	0.350/0.355	0.143/0.163	ns	3	1	0.511	0.727	3.956
TWTZ	2.57	2.40	0.250/0.250	0.273/0.273	0.054/0.088	ns	2	0	0.222	0.041	0.222
GXNN	4.50	3.79	0.621/0.677	0.650/0.660	0.045/−0.025	**	3	2	0.625	0.437	2.375
HNWC1	5.08	3.84	0.484/0.533	0.551/0.577	0.124/0.079	**	15	11	0.911	0.599	3.256
HNWC2	4.08	3.70	0.396/0.440	0.493/0.506	0.202/0.136	**	6	1	0.846	0.533	3.051
GDSZ	4.64	4.02	0.560/0.566	0.600/0.601	0.087/0.060	**	9	4	0.964	2.089	11.364
SCXC	—	—	—	—	—	—	2	1	0.500	1.563	8.500

Pop, population label; *A*, number of alleles; AR, allelic richness; ^R^Ho, observed heterozygosity calculated by the raw data, ^C^Ho, observed heterozygosity calculated by the corrected data; ^R^He, expected heterozygosity calculated by the raw data, ^C^He, expected heterozygosity calculated by the corrected data; ^R^F_IS_, fixation index calculated by the raw data, ^C^F_IS_, fixation index calculated by the corrected data; Nh, no. haplotypes; Nph, number of private haplotypes; Hd, haplotype diversity; π, nucleotide diversity; *k*, average number of nucleotide differences; **indicate significant deviations from HWE at *p *<* *0.01

#### 3.1.2 Genetic variation of *cox I* sequences

Eight populations were used for *cox I* analysis. A total of 544 bp of the *cox I* sequences were generated after final alignment (GenBank accession numbers KY629226‐326). Thirty haplotypes with global values of haplotype diversity and nucleotide diversity and an average number of nucleotide differences were found in the eight populations (total Hd = 0.914, π = 2.313%, *k* = 12.584) (Table 1). The highest levels of haplotype and nucleotide diversities were exhibited in the GDSZ population (Hd = 0.964, π = 2.089%), while the lowest levels were observed in the TWTZ population (Hd = 0.222, π = 0.041%).

### 3.2 Population genetic structure

The overall *F*_ST_ value (*F*_ST_ = 0.259; 95% confidence interval, 0.199–0.330) based on microsatellite data suggested a high level of genetic differentiation among populations. Considering the existence of null alleles, we estimated the corrected pairwise *F*_ST_ value using the ENA algorithm (*F*_ST_^ENA^) across all microsatellite loci and populations, and the results showed that the mean value was 0.265 (95% confidence interval, 0.208–0.339) (for detailed pairwise *F*_ST_ values, see Table 2). The pairwise *F*_ST_ differences based on *cox I* showed significant differentiation in 24 of the 28 population pairs (Table 2).

**Table 2:** Pairwise *F*_ST_ values between all populations based on microsatellite data and *cox I* sequences

Pop	FJTA	FJSM	TWTZ	GXNN	HNWC1	HNWC2	GDSZ	SCXC
FJTA	—	**0.104**	**0.164**	**0.329**	**0.402**	**0.522**	**0.372**	—
FJSM	−0.071	—	**0.088**	**0.283**	**0.362**	**0.482**	**0.333**	—
TWTZ	−0.008	−0.035	—	**0.345**	**0.420**	**0.546**	**0.399**	
GXNN	**0.424**	**0.388**	**0.501**	—	**0.115**	**0.148**	**0.083**	—
HNWC1	**0.283**	**0.253**	**0.349**	**0.190**	—	**0.036**	**0.063**	—
HNWC2	**0.349**	**0.311**	**0.432**	**0.234**	−0.011	—	**0.078**	—
GDSZ	**0.245**	**0.208**	**0.334**	**0.141**	**0.040**	**0.064**	—	—
SCXC	**0.555**	**0.493**	**0.689**	**0.369**	**0.210**	**0.234**	**0.211**	—

Bold indicates significant values (*p* < .05), upper‐right matrix: *F*_ST_^ENA^ values of microsatellite data, lower‐left matrix: *F*_ST_ values of *cox I* sequences.

**Figure 1:**
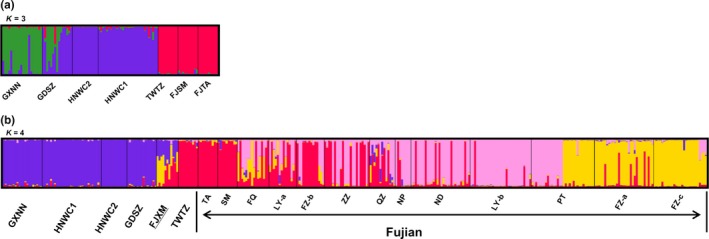
Bar plot of population structure estimates of microsatellite data for *Rhynchophorus ferrugineus* samples in southern China. (a) Samples from new populations in this study; (b) Samples from new populations combined with those from Wang et al. (2015). Each individual is represented by a vertical line that is partitioned into various colored components. Clusters are separated by different colored bars

According to the method described by Evanno et al. (2005), STRUCTURE analyses of seven populations (individuals from Sichuan Province were excluded from microsatellite analysis due to the small sample size) identified three well‐differentiated genetic clusters (*K* = 3, Δ*K* = 582, Fig. S1a). Individuals from Fujian and Taiwan provinces (the FJTA, FJSM and TWTZ populations) were grouped into one cluster, while two populations from Hainan Province (HNWC1 and HNWC2) and one from Guangzhou Province (GDSZ) were grouped into another cluster. Individuals from Guangxi Province (the GXNN population) were grouped into a single cluster (Figure 1a). When microsatellite data from Wang et al. (2015) were included in this analysis, four clusters were found (*K* = 4, Δ*K* = 58, Fig. S1b). Individuals in Fujian Province were divided into three clusters, and Taiwan samples were grouped with one of the Fujian clusters. In addition, the GXNN, HNWC1, HNWC2, and GDSZ populations were grouped into one cluster (Figure 1b).

**Figure 2:**
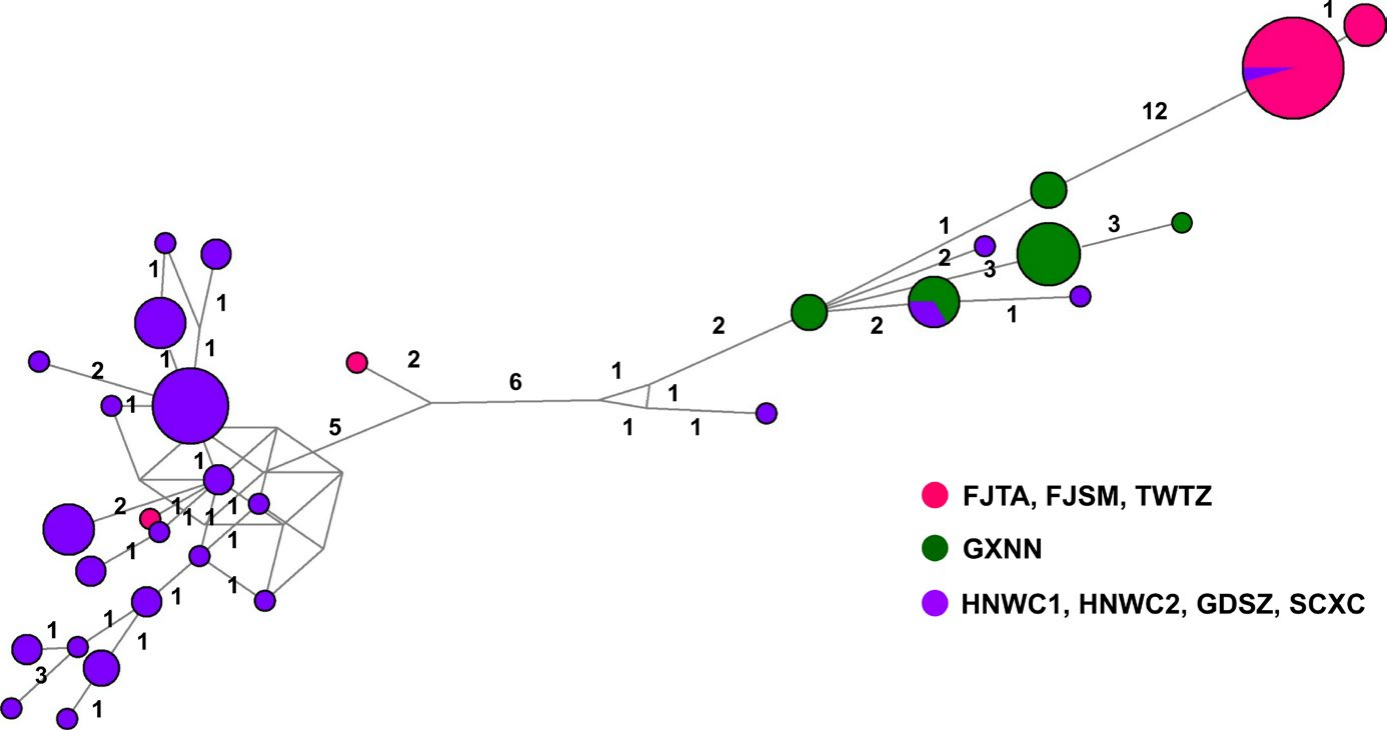
Median Joining network analysis of *cox I* sequences of *Rhynchophorus ferrugineus* in southern China. Each circle represents a haplotype, and sizes are relative to the number of individuals with a specific haplotype. Haplotypes are labeled according to the population of origin. Arabic numbers above the line represent the number of mutant loci

**Figure 3:**
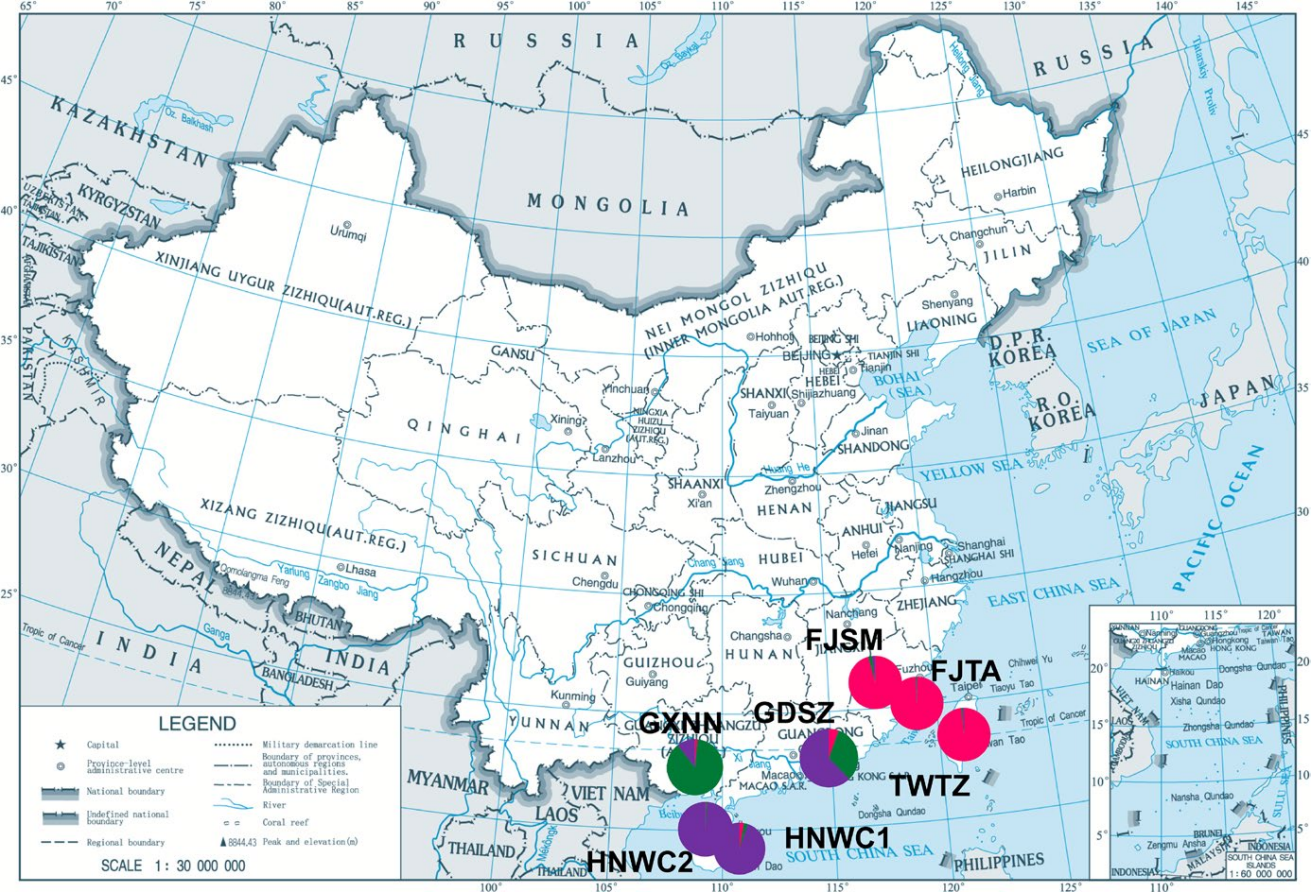
The distribution of seven *Rhynchophorus ferrugineus* populations in southern China based on their STRUCTURE clusters. Note: Redrawn from the website (https://www.travelchinaguide.com/map/china_map.htm#) using Adobe Illustrator Artwork 17.0 software

Median joining (MJ) network analysis of *cox I* sequences from eight populations showed similar cluster results as shown by the microsatellite analysis (Figure 2). To clearly show the genetic structure predicted by the two methods, different populations were assigned colors according to the cluster results of the STRUCTURE software; individuals in the SCXC population were clustered in the same group with individuals from the HNWC1 and HNWC2 populations. A significant distribution of haplotype clusters of the RPW in southern China was observed (Figure 3).

**Figure 4:**
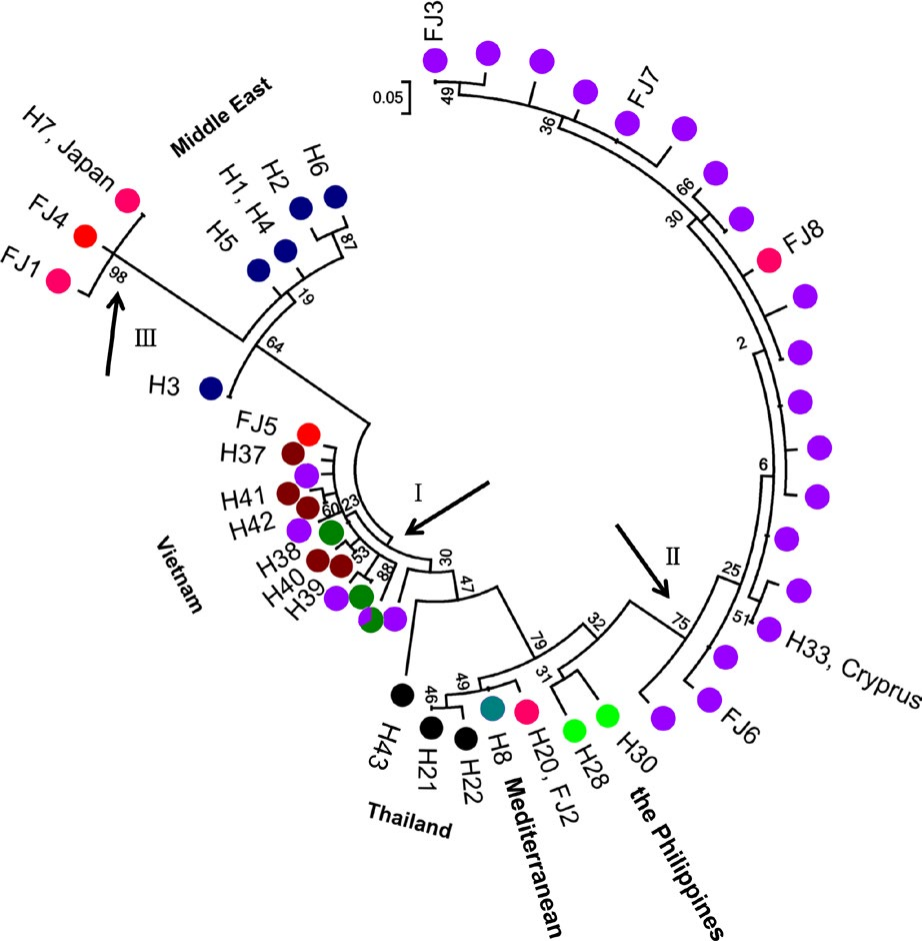
Unrooted maximum‐likelihood tree showing the phylogenetic relationship of *Rhynchophorus ferrugineus* populations in China and other countries based on haplotypes. Color schemes of new haplotypes are shown according to Figure 2. Branch I contains haplotypes in GXNN, GDSZ, and SCXC populations; haplotypes of HNWC1 and HNWC2 populations and one FJTA individual are contained in branch II; FJTA, FJSM, and TWTZ haplotypes are contained in branch III. Other haplotypes are as follows: FJ1–FJ8 (Fujian, Wang et al., 2015), H1–H6 (the Middle East, El‐Mergawy et al., 2011), H21–H22, H43 (Thailand, Rugman‐Jones et al., 2013), H28, H30 (the Philippines, Rugman‐Jones et al., 2013), and H37–H42 (Vietnam, Rugman‐Jones et al., 2013)

We combined our *cox I* sequences with 29 sequences obtained from GenBank (Table S3) to construct the phylogenetic tree. The maximum‐likelihood tree of haplotype data showed that individuals in branch I (the GXNN, GDSZ, and SCXC populations) were close to those from Vietnam; haplotypes in branch II (the HNWC1 and HNWC2 populations and some GDSZ individuals) were close to those from the Philippines, Thailand, and the Mediterranean; and haplotypes in branch III (the FJTA, FJSM, and TWTZ populations) were closely related to those from Japan and the Middle East (Figure 4).

**Figure 5:**
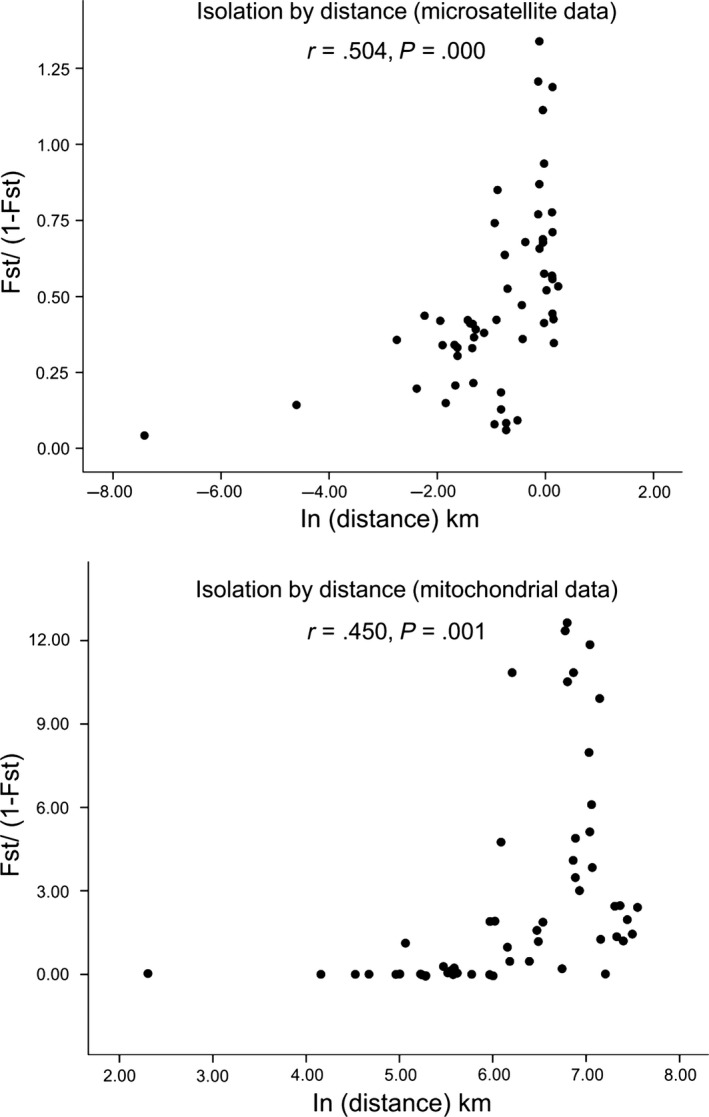
Scatter plots of genetic isolation by geographical distance and genetic distance among *Rhynchophorus ferrugineus* populations using microsatellite and mitochondrial sequences

Population genetic variances were tested according to the genetic structure clusters. Three‐level hierarchical AMOVA analysis of microsatellite markers and *cox I* supported the results of Bayesian cluster and network analyses, with three genetic clusters (*df* = 2, percentage of variation = 28.05%, *p *=* *.01857; *df* = 2, percentage of variation = 69.66%, *p *=* *.00293 (Table 3)). The IBD test produced an *r* value of 0.504 for microsatellite data (*p *=* *.000) and 0.450 for mitochondrial genes (*p *=* *.001) when the four previous Fujian populations (FZ‐a, FZ‐c, ZZ, LY‐b) (for details, see Wang et al., 2015) were added to the analysis, suggesting a significant correlation between genetic and geographical distances among the different populations (Figure 5).

**Table 3:** AMOVA results of *Rhynchophorus ferrugineus* populations based on microsatellite data and *cox I* sequences

	Source of variation	*df*	SS	Variance components	% Variation	Fixation index
Microsatellite	Among three clusters	2	194.408	1.33315 Va	28.05	FCT = 0.28050*p* = .01857 ± .00459
	Among populations within clusters	4	37.791	0.22455 Vb	4.72	FSC = 0.06567*p* ≤ .0000
	Within seven populations	209	667.774	3.19509 Vc	67.23	FST = 0.32775*p* ≤ .0000
*cox I* sequences	Among clusters	2	396.311	6.10852 Va	69.66	FCT = 0.75749*p* = .00293 ± .00164
	Among populations within clusters	5	44.405	0.60160 Vb	6.86	FSC = 0.22615*p* = .00000
	Within populations	93	191.443	2.05852 Vc	26.48	FST = 0.76527*p* = .00000

### 3.3 Gene flow and demographic history

The gene flow values suggested the presence of variable levels of gene flow between populations (Table S4). The highest level of gene flow observed was between the HNWC1 and HNWC2 populations (6.618). Intermediate gene flow levels (*N*_m_ > 1) were observed between three population pairs (FJTA vs. FJSM, FJTA vs. TWTZ, and FJSM vs. TWTZ) and between the population pairs (GXNN vs. HNWC1, GXNN vs. HNWC2, GXNN vs. GDSZ, GDSZ vs. HNWC1, and GDSZ vs. HNWC2), indicating small genetic differentiation between these population pairs. The microsatellite data showed low levels of gene flow between the south‐eastern group (the FJTA, FJSM, and TWTZ populations) and the southern group (the GXNN, GDSZ, HNWC1, and HNWC2 populations). Mitochondrial sequences showed a low average estimate of gene flow (*N*_m_ = 0.11) throughout the populations.

The result from the bottleneck effect test revealed significant heterozygote excess in the GXNN population based on the IAM (*p *=* *.00003) and TPM (*p *=* *.00131) models and in the HNWC1 population according to the IAM model (*p *=* *.03925). Additionally, the test was nonsignificant for all populations under the SMM model (Table S5).

Together with Tajima's D (2.099, *p *=* *.933) and Fu's Fs (−0.563, *p *=* *.604) tests, mismatch distribution analysis showed a multimodal mismatch graph when all populations were considered as a single group (Fig. S2a), indicating no evidence of recent demographic expansion. These results were confirmed by a Bayesian skyline plot (BSP) analysis based on mitochondrial data (Fig. S2b).

## 4 DISCUSSION

### 4.1 Genetic diversity of RPW populations in southern China

By genotyping different populations from the major RPW outbreak centers in China using microsatellite markers, we determined that genetic variation was generally low within the southern China populations. These results were consistent with those previously found for the RPW in Fujian Province (Wang et al., 2015). The average number of alleles per locus per location was high in populations from Hainan (HNWC1 and HNWC2), intermediate in populations from Guangdong (GDSZ) and Guangxi (GXNN) provinces, and relatively low in populations from Fujian (FJTA and FJSM) and Taiwan (TWTZ) provinces in our study. Both microsatellite and *cox I* data revealed that the lowest genetic diversity was observed in individuals from Taiwan Province (TWTZ). The insular location of Taiwan may contribute to the low level of genetic variation. A narrow ocean barrier coupled with a small population size may dramatically influence the genetic diversity. Wu et al. (2015) reported that changes in ocean barriers have influenced gypsy moth population structure, which was verified by the distribution of haplotypes and analysis of alleles. The successful invasion of this species does not appear to depend on high levels of genetic variation. The genetic diversity of an invasive species is expected to decrease with range expansion and colonization of new areas via the founder effect or by a genetic bottleneck (Dlugosch & Parker, 2008; Ramachandran et al., 2005). Low genetic diversity in the TWTZ population is not due to bottlenecks, however, as a nonsignificant bottleneck signature was observed in this population (IAM, *p *=* *.94922; TPM, *p *=* *.99194; SMM, *p *=* *.99658). Founder effects produced by genetic drift occurring in a small number of individuals probably contributed to the low genetic diversity in the TWTZ population.

Our results showed that four of the seven new populations significantly deviated from HWE and that three populations (HNWC1, HNWC2, and GDSZ) displayed significant heterozygote deficiency and positive *F*_IS_ values, indicating that high inbreeding existed in these populations. This finding might be the result of nonrandom mating caused by the gregarious habits and generation overlap of RPW populations, which increase the probability of inbreeding. Studies of *Frankliniella occidentalis* showed that high inbreeding significantly caused a deviation from HWE (Yang, Sun, Xue, Li, & Hong, 2012). Heterozygote excess was found in the GXNN population with a negative *F*_IS_ value, which is consistent with the result of the heterozygote excess test using BOTTLENECK software (IAM, *p *=* *.00003 and TPM, *p *=* *.00131). We therefore deduced that individuals in the GXNN population had experienced a bottleneck effect and did not meet the criteria for HWE.

High haplotype diversity (Hd > 0.5) was present in most populations, especially in individuals from the two southernmost provinces, Hainan (HNWC1 and HNWC2) and Guangdong (GDSZ), indicating that these populations have experienced more propagule pressure. Multiple introduction or repeated introduction events will enhance diversity among invasive populations. The local climate seems to play a role in the expansion process of invasive species. Located in the tropical zone, with relatively high temperatures and rainfall amounts and a wide diversity of potential hosts, Hainan is considered an optimal habitat for the RPW. The high level of ecosystem invasibility of Hainan Island promotes colonization by invasive populations. The RPW has infested many coconut palms in Hainan and has had a serious economic impact on the island. Shenzhen City has the most ports in China and is also an important border city in Guangdong Province; the (international) trade of palms from native or infected areas to new areas across this province increased the possibility of infection by invasive species. Based on the palm transportation records in China, frequent human‐aided dispersal events occurred in these two provinces (Li & Yao, 2006; Xu, 2005; Zhang, 2007). We can therefore deduce that individuals from these two provinces have been subjected to disproportionate invasion pressures, which enhance population diversity and consequently have resulted in an excess number of haplotypes during population colonization.

### 4.2 Population genetic structure and expanding events

The pairwise *F*_ST_ values based on microsatellite and *cox I* data showed a clear genetic differentiation among populations, suggesting that large genetic divergences existed. Significant divisions were observed in studied populations from Fujian/Taiwan and in those from Guangdong/Hainan/Sichuan or from Guangxi according to the results of the NETWORK software analysis. Overall, thirty haplotypes were found in the studied populations, 22 of which were unique. Combining the present haplotypes with those from previous studies (Abulyazid et al., 2002; El‐Mergawy et al., 2011; Rugman‐Jones et al., 2013; Wang et al., 2015), the distribution of all of the haplotypes can be divided into three distinct clades. Firstly, individuals along the south‐eastern coast of China (branch III: FJTA, FJSM, and TWTZ) are presumably close to the H7 haplotype found in Japan (El‐Mergawy et al., 2011). As reported by Rugman‐Jones et al. (2013), the H7 haplotype seems to have originated from the north‐western part of its native range, perhaps India, Bangladesh, or Myanmar. Additionally, haplotypes from the Middle East were grouped with those from India and Sri Lanka in the study by Rugman‐Jones et al. (2013). Secondly, samples from Guangxi (GXNN), Guangdong (GDSZ), and Sichuan (SCXC) provinces (branch I) share a close relationship with those from Vietnam. Thirdly, the Hainan samples (HNWC1 and HNWC2), together with H33, are clustered into branch II. The H33 haplotype found in Cyprus is closely related to haplotypes from the Philippines (Rugman‐Jones et al., 2013). Based on *cox I* sequences taken from previous studies (El‐Mergawy et al., 2011; Rugman‐Jones et al., 2013; Wang et al., 2015), the most likely native sources of RPW in southern China are India, the Philippines, and Vietnam.

The microsatellite structure of seven new populations showed that individuals from Guangdong Province shared high levels of gene flow with those from Guangxi and Hainan provinces (Figure 1a), which indicated that there were mixed introductions or multiple introductions of the RPW. Our investigation revealed that the ornamental palm plants in Fairy Lake Botanical Garden, Shenzhen & Chinese Academy of Sciences (our sample location), were introduced from several different palm parks or nursery gardens from other provinces (Guangxi, Fujian, Yunnan, Hainan, etc.) or countries. However, the combined results showed that the genetic compositions of the GDSZ and GXNN populations were similar to those from Hainan Province. Compared with the newly studied populations, samples from Fujian Province were collected from markedly more sites, which may have resulted in an asymmetric genetic structure. According to the genetic structure analysis of the RPW in China, it can be stated that the weevils from Taiwan and Fujian provinces are derived from different native sources than those from Hainan Province and other populations in mainland China.

The limitation of gene flow associated with the genetic differentiation among clusters gave rise to strong genetic structuring. A significant correlation was detected between genetic and geographical distances. Although the Taiwan Strait is between Taiwan and Fujian provinces and the Qiongzhou Strait is between Guangdong and Hainan provinces, gene flow between the populations was high, indicating that geographical barriers were not a main factor that prevented the distribution of the RPW in these regions. Furthermore, the Hercynian construction and the Maritime Silk Road construction may considerably increase human‐aided transportation, which will enhance immigration between these regions. According to these results, it is possible to hypothesize that based on a broad perspective, geographical distances have historically played a very important role in the large‐scale genetic structure of the RPW in southern China, although anthropogenic activities cannot be ignored.

In summary, after testing the genetic characteristics of the RPW in southern China, we found strong genetic structure, which indicated a significant connection between the geographical distribution and anthropogenic activities. More than one haplotype detected in one population revealed that multiple introduction events from other infected areas (including native or invasive regions) to China played a major role in the distribution of haplotypes in southern China. From palm plants and offshoot transportation, we can deduce that the RPW in China originated from different populations and followed various paths. The origin sources of this pest in southern China are likely from India, the Philippines, and Vietnam. To some extent, the construction of city gardens, which promoted palm plant transportation in China, widely influenced the distribution of the RPW. On the other hand, the successful invasion of the RPW in southern China is probably due to its biological attributes together with the existence of numerous suitable habitats and climates across southern China (Qin et al., 2009). The cryptic behavior, egg deposition inside plant tissue, and polyphagous nature (feeding on 40 palm species, even sugarcane [Nirula, 1956; Lever, 1969; Esteban‐Duran et al., 1998; OJEU, 2008; Anonymous, 2013]), make detection difficult and facilitate the invasion of the RPW to new environments. Additionally, because of its high fecundity (total lifetime fecundities range from 58 to 531 progeny per female) (Wattanapongsiri, 1966), high population growth potential and survival rate (Qin et al., 2009; Rahalkar, Harwalkar, & Rananvare, 1972), RPW can easily become established in new areas. Other population colonization factors such as founder effects and bottleneck effects would also influence their genetic background.

## CONFLICT OF INTEREST

None declared.

## AUTHOR CONTRIBUTIONS

All the authors in this manuscript contributed to the work. Y.M.H. and G.H.W. conceived and designed the research; X.Z., J.Z., Z.M.C., and G.H.W. collected RPW samples and generated and analyzed the data; and J.L.L. and G.H.W. conducted the experiments and wrote the manuscript. All authors read and approved the manuscript.
