# Supplementary material for: Strong population genetic structure of an invasive species, Rhynchophorus ferrugineus (Olivier), in southern China
Source: Ecol Evol. 2017 Nov 7;7(24):10770–81. doi: 10.1002/ece3.3599 (PMC5743574; doi:10.1002/ece3.3599)
Supplement: Supplementary file 1 [file ECE3-7-10770-s001.doc]

**Strong population genetic structure of an invasive species,** ***Rhynchophorus ferrugineus* (Olivier), in southern China**

Guihua Wang^1,2^, Youming Hou^1,2^, Xiang Zhang^1,2^, Jie Zhang^1,2^, Jinlei Li^1,2^, Zhiming Chen^3^

^1^ *State Key Laboratory of Ecological Pest Control for Fujian and Taiwan Crops, Fujian Agriculture and Forestry University, Fuzhou 350002, Fujian, China,* ^2^*Fujian Province Key Laboratory of Insect Ecology, College of Plant Protection*, *Fujian Agriculture and Forestry University, Fuzhou, 350002, Fujian, China and* ^3^*Fuzhou Entry-Exit Inspection & Quarantine Bureau of P.R.C*

Correspondence:

Prof. Dr. Youming Hou

Department of Plant Protection,

Fujian Agriculture and Forestry University, Fuzhou, Fujian 350002, China

Phone: +86-591-83768654

Fax: +86-591-83768654

E-mail: ymhou@fafu.edu.cn

**Table S1** Sampling information of *Rhynchophorus ferrugineus* populations

| **Pop** | **Location** | **host plant (if known)** | **Collection date** | **Longitude/ Latitude** | **n** |
| --- | --- | --- | --- | --- | --- |
| **FJTA** | Tongan, Fujian | *Phoenix canariensis* | 18-Jun-2013 | E118°09′, N24°43′ | 10 |
| **FJSM** | Sanming, Fujian | *P. canariensis* | 2-Jul-2013 | E117°21′, N25°56′ | 10 |
| **TWTZ** | Taizhong, Taiwan | *Areca catechu* | 16-Apr-2014 | E120°40′, N24°09′ | 10 |
| **GXNN** | Nanning, Guangxi | *P. sylvestris* | 15-May-2013 | E108°21′, N22°48′ | 20 |
| **HNWC1** | Wenchang, Hainan | *Cocos nucifera* | 6-Sept-2013 | E110°47′, N19°32′ | 30 |
| **HNWC2** | Wenchang, Hainan | *C. nucifera* | 15-Sept-2014 | E110°45′, N19°30′ | 13 |
| **GDSZ** | Shenzhen, Guangdong | *Bismarckia nobilis* | 8-Jul-2014 | E114°09′, N22°34′ | 15 |
| **SCXC** | Xichang, Sichuan | Palm plants | 30-Aug-2013 | E102°11′, N27°59′ | 4 |

Pop, population label; n, number of individuals

**Table S2** Primer sequences and characteristics of the 14 microsatellite loci used in this study

| Locus | Primer sequence 5′-3′ | Repeat motif | Fluorescent label | Allele size | Tm (°C) |  |
| --- | --- | --- | --- | --- | --- | --- |
| P1A3 | F:CACCTTTAATAGTTCTTCTGACAT  R:AAAAGACAAGGAAATCCACA | (GT)_14_ | FAM | 182-230 | 56.0 | Wang (2015) |
| P1C11 | F: TCCTGCGAACAAAGAGAAA  R: GCAAAAATCACTCGGACA | (TG)_10_ | HEX | 185-199 | 59.0 | Wang (2015) |
| P4D8 | F: AGGTGATTTTGGGCTCTTTT  R: AGCATATTCGTATCCGTTAGAG | (GT)_11_ | ROX | 110-118 | 53.0 | Wang (2015) |
| P1E2 | F: CATTGATGTTGATTTTCGATT  R: ACCATGAGATCGGCTGTTT | (GA)_20_ | FAM | 114-178 | 55.0 | Wang (2015) |
| P1C8 | F: CCGATCCAATTCCCTAAA  R: CGTTCGGTTTACGTGTCC | (GT)_9_ | HEX | 181-210 | 55.0 | Wang (2015) |
| P2F6 | F: CGTGGGACCTTATTCGTG  R: CTTTCCGTCTAACTTTCCTTTT | (GT)_13_ | ROX | 118-237 | 52.0 | Wang (2015) |
| P3G8 | F: GATCCGACCGAAACTAACC  R: CAATCCTCACCAAAACAAAA | (GT)_18_ | FAM | 161-179 | 51.0 | Wang (2015) |
| P2F11 | F: TGACTCATGGATTTTGTCATT  R: GGCAACTCTTTCGCACTTT | (AG)_10_ | HEX | 247-253 | 56.0 | Wang (2015) |
| P2F8 | F: GCCTTAGACTTTGTCCTACCC  R: ATTCCTTATTCGCCTGACTT | (TG)_8_ | FAM | 119-257 | 50.0 | Wang (2015) |
| P3A8 | F: ATACGCCGCACAAAAACA  R: CGAAACAAAGACCAGGAAAA | (GT)_9_ | ROX | 262-266 | 51.0 | Wang (2015) |
| P3E5B | F: ATTCCTTGCGTCGTATTTGT  R: GCTCGAATTGCGTCCTC | (GT)_8_ | FAM | 201-209 | 56.0 | Wang (2015) |
| P1F12 | F: CGAGATGAATGGGAAGG  R: CTAAACACCGAGCCGAACT | (TG)_8_ | HEX | 178-182 | 57.0 | Wang (2015) |
| P1F8 | F:TTAGATGCTACGTGATAGAAGAC  R: CAGCCGGTCCATACACA | (GA)_27_ | ROX | 118-163 | 65.0 | Wang (2015) |
| P3A10 | F: TCAGACCCTTTCGCTCTTAC  R: CAACAAAAGCCGTTCTCTAC | (GT)_12_ | ROX | 164-225 | 53.0 |  |

**Table S3** Information of sequences used for haplotype phylogenetic analysis

| **haplotype** | **collection site** | **accession number** |
| --- | --- | --- |
| **H1** | Middle-East, El-Mergawy (2011) | GU581582 |
| **H2** | Middle-East, El-Mergawy (2011) | GU581549 |
| **H3** | Middle-East, El-Mergawy (2011) | GU581548 |
| **H4** | Middle-East, El-Mergawy (2011) | GU581545 |
| **H5** | Middle-East, El-Mergawy (2011) | GU581539 |
| **H6** | Middle-East, El-Mergawy (2011) | GU581526 |
| **H7** | Japan, EI-Mergawy (2011) | GU581517 |
| **H8** | Mediterranean, El-Mergawy (2011) | GU581319 |
| **H20** | Aruba, Rugman-Jones (2013) | KF311433 |
| **H21** | Thailand, Rugman-Jones (2013) | KF311474 |
| **H22** | Thailand, Rugman-Jones (2013) | KF311510 |
| **H28** | the Philippines, Rugman-Jones (2013) | KF311399 |
| **H30** | the Philippines, Rugman-Jones (2013) | KF311392 |
| **H33** | Cyprus, Rugman-Jones (2013) | KF311461 |
| **H37** | Vietnam, Rugman-Jones (2013) | KF311509 |
| **H38** | Vietnam, Rugman-Jones (2013) | KF311502 |
| **H39** | Vietnam, Rugman-Jones (2013) | KF311508 |
| **H40** | Vietnam, Rugman-Jones (2013) | KF311506 |
| **H41** | Vietnam, Rugman-Jones (2013) | KF311507 |
| **H42** | Vietnam, Rugman-Jones (2013) | KF311501 |
| **H43** | Thailand, Rugman-Jones (2013) | KF311525 |
| **FJ1** | Fujian, China, Wang (2015) | KF413063 |
| **FJ2** | Fujian, China, Wang (2015) | KF413064 |
| **FJ3** | Fujian, China, Wang (2015) | KF413065 |
| **FJ4** | Fujian, China, Wang (2015) | KF413066 |
| **FJ5** | Fujian, China, Wang (2015) | KF413067 |
| **FJ6** | Fujian, China, Wang (2015) | KF413068 |
| **FJ7** | Fujian, China, Wang (2015) | KF413069 |
| **FJ8** | Fujian, China, Wang (2015) | KF413070 |

**Table S4** Gene flow among different populations based on microsatellite data

|  | **FJTA** | **FJSM** | **TWTZ** | **GXNN** | **HNWC1** | **HNWC2** |
| --- | --- | --- | --- | --- | --- | --- |
| **FJSM** | **2.1585** |  |  |  |  |  |
| **TWTZ** | **1.2791** | **2.5941** |  |  |  |  |
| **GXNN** | 0.5099 | 0.6346 | 0.4740 |  |  |  |
| **HNWC1** | 0.3720 | 0.4402 | 0.3457 | **1.9334** |  |  |
| **HNWC2** | 0.2292 | 0.2685 | 0.2078 | **1.4403** | **6.6181** |  |
| **GDSZ** | 0.4226 | 0.5003 | 0.3761 | **2.7766** | **3.6994** | **2.9592** |

Bold numbers indicated gene flow >1

**Table S5** One-tailed P-values for the Wilcoxon test for heterozygosity excess under three mutational models

| **populations** | **Heterozygosity excess P-values** | | |
| --- | --- | --- | --- |
|  | IAM | TPM | SMM |
| **FJTA** | 0.25928 | 0.68896 | 0.80981 |
| **FJSM** | 0.96619 | 0.99899 | 0.99991 |
| **TWSZ** | 0.06763 | 0.27081 | 0.82126 |
| **GXNN** | **0.00003** | **0.00131** | 0.31287 |
| **HNWC1** | **0.03925** | 0.50000 | 0.96619 |
| **HNWC2** | 0.39343 | 0.90454 | 0.99799 |
| **TWTZ** | 0.94922 | 0.99194 | 0.99658 |


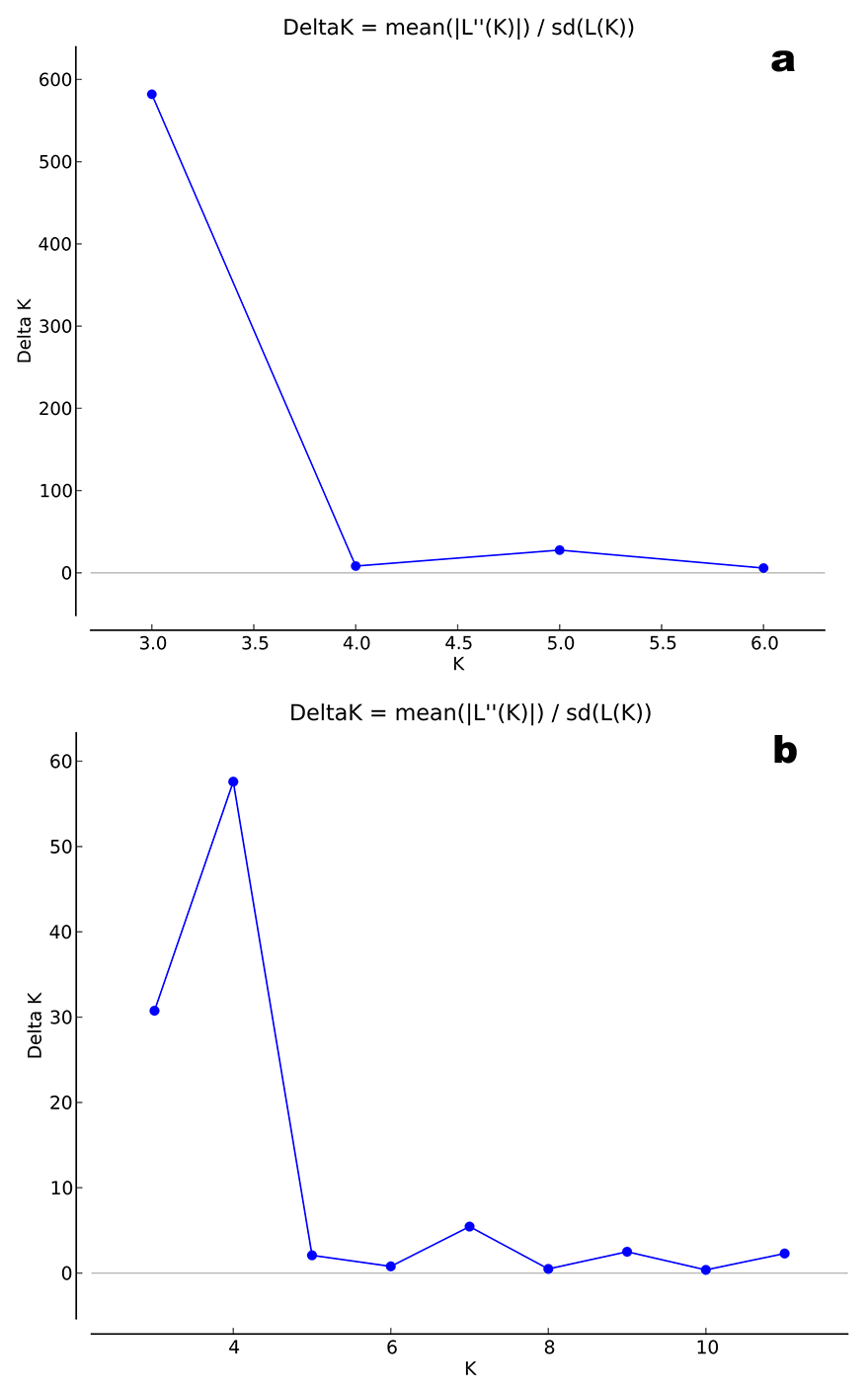


**Figure S1** The graph of the number of the inferred clusters (K) using Evanno’s delta (K) method. a: Samples from new populations in this study; b: Samples from new populations combined with those from Wang *et al*. (2015).


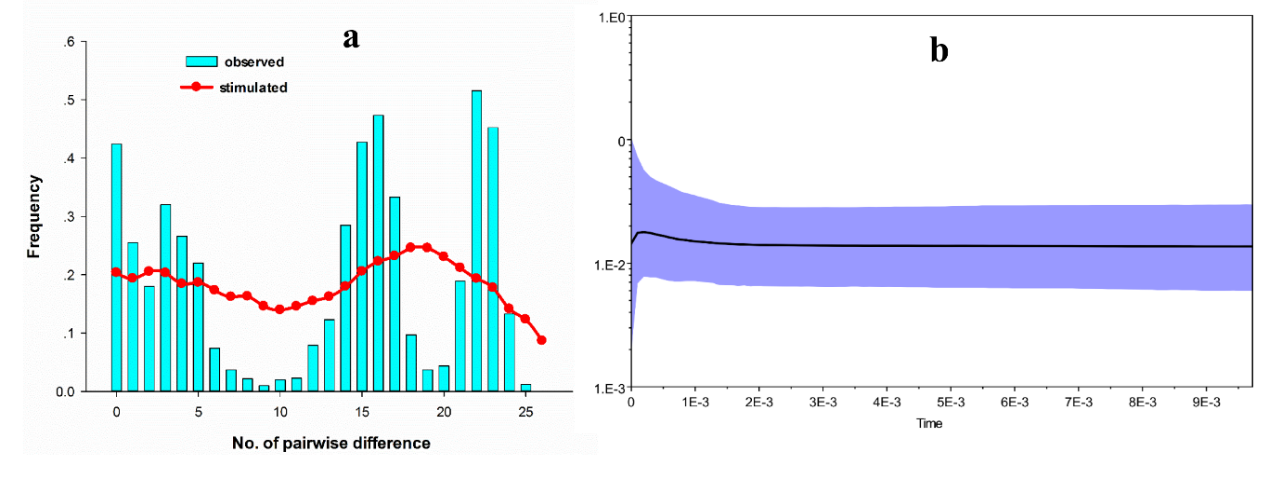


**Figure S2** Mismatch distribution analysis as well as bayesian skyline plot (BSP) analysis indicated no evidence of recent demographic expansion.
